# Host cell remodeling by pathogens: the exomembrane system in *Plasmodium*-infected erythrocytes

**DOI:** 10.1093/femsre/fuw016

**Published:** 2016-08-29

**Authors:** Emma S. Sherling, Christiaan van Ooij

**Affiliations:** 1 The Francis Crick Institute, Mill Hill Laboratory, Mill Hill, London NW7 1AA, UK; 2 Laboratory of Malaria and Vector Research, National Institute of Allergy and Infectious Diseases, National Institutes of Health, Rockville, MD 20852, USA

**Keywords:** malaria, host–parasite interaction, pathogenesis, exomembrane system, plasmodium

## Abstract

Malaria is caused by infection of erythrocytes by parasites of the genus *Plasmodium*. To survive inside erythrocytes, these parasites induce sweeping changes within the host cell, one of the most dramatic of which is the formation of multiple membranous compartments, collectively referred to as the exomembrane system. As an uninfected mammalian erythrocyte is devoid of internal membranes, the parasite must be the force and the source behind the formation of these compartments. Even though the first evidence of the presence these of internal compartments was obtained over a century ago, their functions remain mostly unclear, and in some cases completely unknown, and the mechanisms underlying their formation are still mysterious. In this review, we provide an overview of the different parts of the exomembrane system, describing the parasitophorous vacuole, the tubovesicular network, Maurer's clefts, the caveola-vesicle complex, J dots and other mobile compartments, and the small vesicles that have been observed in *Plasmodium*-infected cells. Finally, we combine the data into a simplified view of the exomembrane system and its relation to the alterations of the host erythrocyte.

## Abstract (precis)

Plasmodium parasites remodel the host erythrocyte in various ways, including the formation of several membranous compartments, together referred to as the exomembrane system, within the erythrocyte cytosol that together are key to the sweeping changes in the host cell.

## INTRODUCTION

Soon after the discovery of the *Plasmodium* parasite in the late 1800s as an intracellular pathogen of erythrocytes that causes malaria (Laveran 1880), it became clear that the parasite induces sweeping changes within the host cell. Staining of infected erythrocytes with Romanowsky and Giemsa stains revealed punctate staining patterns that were absent from uninfected erythrocytes (Schüffner 1899; Maurer 1900, 1902; Stephens and Christophers 1903). It also became clear that different *Plasmodium* species induce different changes, as indicated by their different staining patterns (Table 1). The development of staining protocols and the identification of the different staining patterns have been described in detail (Lanzer *et al*. 2006; Wickert and Krohne 2007).

**Table 1.** The exomembrane systems of various *Plasmodium* species.

			EM				
				Plasma membrane			
Parasite type	Species	Light microscopy	Cytoplasmic clefts	Excrescences	Caveola	Caveola-vesicle complex	Reference
Vivax type	*Plasmodium vivax*	Schüffner's dots	+	−	+	+	Aikawa, Miller and Rabbege (1975)
	*Plasmodium simium*	Schüffner's dots	+	−	+	+	Sterling *et al*. (1975)
	*Plasmodium cynomolgi*	Schüffner's dots	+	−	+	+	Aikawa, Miller and Rabbege (1975)
Ovale type	*Plasmodium ovale*	Schüffner's dots	+	+	+	+	Matsumoto, Matsuda and Yoshida (1986)
	*Plasmodium simiovale*	Schüffner's dots	+	±	+	+	Aikawa, M., Miller, L.H., Rabbage, J., unpublished
	*Plasmodium fieldi*	Schüffner's dots	+	±	+	+	Aikawa, M., Miller, L.H., Rabbage, J., unpublished
Falciparum type	*Plasmodium falciparum*	Maurer's clefts	+	+^a^	−	−	Luse and Miller (1971)
	*Plasmodium fragile*	Faint stippling	+	+	+	−	Fremount and Miller (1975)
	*Plasmodium coatneyi*	Maurer's clefts	+	+^a^	+	−	Rudzinska and Trager (1968)
							
Malariae type	*Plasmodium malariae*	Ziemann's stippling	+	+^a^	−	−	Smith and Thekson (1970)
	*Plasmodium brasilianum*	Ziemann's stippling	+	+^a^	−	−	Sterling, Aikawa and Nussenzweig (1972)
Others	*Plasmodium knowlesi*	Sinton and Mulligan's stippling	+	−	+	−	Miller, Fremount and Luse (1971)

a Excrescences are on erythrocytes infected by asexual forms and those infected by gametocytes of *P. malariae* and *P. brasilianum*, but they are only on erythrocytes with asexual forms of *P. facliparum* and *P. coatneyi*. Reproduced from Aikawa, Miller and Rabbege (1975) with permission from the American Society for Investigative Pathology. This content is not covered by the terms of the Creative Commons licence of this publication. For permission to reuse, please contact the rights holder.

There are five species of *Plasmodium* parasites that infect humans: *Plasmodium falciparum*, *P. vivax*, *P. ovale*, *P. malariae* and *P. knowlesi*, although the latter is a zoonotic infection and not spread from human to human. Of these, *P. vivax* is the most widespread globally, but nearly all the fatalities (most recent estimates indicate around 600 000 fatalities owing to malaria annually; WHO 2014) are the result of infection with *P. falciparum*. After infection of a host, the parasites undergo a period of replication in the liver. Infectious progeny, called merozoites, are subsequently released into the blood stream, where they infect either erythrocytes and/or reticulocytes. It is this stage that leads to clinical malaria. Despite differences in host cell preference, length of the intraerythrocytic lifecycle and the number of progeny produced, the basics of the intraerythrocytic lifecycle of these species are very similar: after binding to an erythrocyte, the parasite invades the host cell by pushing itself into the host cell, causing an indentation in the membrane of the host. This indentation grows until the parasite is completely surrounded by membrane, at which point the membrane pinches off behind the invading merozoite, leaving the parasite free in the cytosol, but still surrounded by membrane. The compartment surrounding the parasites is referred to as the parasitophorous vacuole (PV) and the membrane that delineates the PV is referred to as the parasitophorous vacuole membrane (PVM). For the first half of the intraerythrocytic cycle (which in total lasts between 24 and 72 h, depending on the species), the parasite remains in the ring form, which displays low metabolic activity and changes little morphologically. The parasite then transitions to the trophozoite (feeding) stage, in which it becomes more metabolically active and grows rapidly. DNA synthesis and nuclear division, but not cytokinesis, take place until ultimately the parasite becomes a schizont with the onset of nuclear division; the total number of nuclei formed in a mature schizont ranges from 8 to 32, depending on the species (Bannister *et al*. 2000). Rapid cytokinesis then allows budding of individual nuclei and the organelles required for invasion to form individual merozoites, which are finally released from the host in a process referred to as egress (Blackman and Carruthers 2013) and subsequently invade new erythrocytes.

Uninfected erythrocytes have a low metabolic activity (Gronowicz, Swift and Steck 1984; Chasis *et al*. 1989) and do not synthesize phospholipids. After invasion by a parasite, the host erythrocyte undergoes numerous transformations, including changes in its rigidity, adhesiveness and permeability to nutrients. During *in vitro* growth, the parasite requires many nutrients from outside the cell, including sugars, amino acids, purines, vitamins, choline and fatty acids (Divo *et al*. 1985; Asahi *et al*. 2005; Asahi 2009). As an example, glucose uptake increases over 50-fold in infected erythrocytes as compared to uninfected erythrocytes (Scheibel, Adler and Trager 1979; Roth *et al*. 1982; Asahi *et al*. 1996; Lang-Unnasch and Murphy 1998). The adhesiveness of the infected erythrocyte also increases, promoting survival of the parasite in the host by sequestering infected erythrocytes in the periphery and thereby preventing clearance in the spleen (Langreth and Peterson 1985).

Underlying all these changes are parasite proteins that are exported from the parasite into the host erythrocyte. For example, the increased adhesiveness of the infected erythrocyte to endothelial cells that removes mature parasites from the circulation (Bignami and Bastianelli 1889; Miller 1969) is mediated by PfEMP1 (Aley, Sherwood and Howard 1984; Leech *et al*. 1984b; Baruch *et al*. 1995; Smith *et al*. 1995; Su *et al*. 1995), a parasite protein that is transported to the surface of the host erythrocyte, where it is part of the knob complex, the structure that is required for the binding of PfEMP1 to the endothelium, formed together with KAHRP (Leech *et al*. 1984a; Crabb *et al*. 1997) and likely many other proteins (Oberli *et al*. 2014, 2016). The increased uptake of solutes such as glucose and hypoxanthine is potentially regulated by Clag 3.1, a protein that forms the *Plasmodium* surface anion channel (PSAC) or that acts on a host protein to increase nutrient uptake (Nguitragool *et al*. 2011), and the changes in deformability are mediated by many proteins that bind to and modify the host cytoskeleton (Maier *et al*. 2008, 2009; Prajapati and Singh 2013).

However, the most striking change in the infected cell observed in Giemsa-stained smears is the one that was identified over a hundred years ago: the *de novo* formation of an exomembrane system in the infected erythrocyte that is detected by Romanowsky and Giemsa staining. This collection of membranous compartments that are not present in uninfected cells first came into focus with the application of electron microscopy (EM) to the study of infected erythrocytes, starting with the first EM images of thin sections of erythrocytes infected with *P. berghei* and *P. knowlesi* (Fulton and Flewett 1956) (which finally laid to rest doubts about of the intracellular localization of the parasites), followed by the groundbreaking studies of Maria Rudzinska, William Trager and Masamichi Aikawa, among others. These revealed that the parasite resides within an intraerythrocytic membrane-bound compartment, the PV, the membrane of which separates the parasite from the cytosol of the erythrocyte (Ladda, Arnold and Martin 1966), and that the infected cell contains various other membranous compartments, including the Maurer's clefts (MCs) (Trager, Rudzinska and Bradbury 1966; Aikawa 1971; Hanssen *et al*. 2008a).

The development of an *in vitro* culture system (Trager and Jensen 1976) allowed more detailed investigation of infected erythrocytes, while the development of transfection technologies for genetic manipulation of the parasite (Goonewardene *et al*. 1993; Wu *et al*. 1995; Wu, Kirkman and Wellems 1996; Crabb *et al*. 1997; Sultan *et al*. 1997) provided the opportunity to alter and delete genes and examine the contribution of individual proteins. The expression of fluorescent proteins further allowed the unraveling of the pathways through which parasite proteins are transported into the host erythrocyte (VanWye and Haldar 1997; de Koning-Ward *et al*. 1998). Together with the use of various fluorescent membrane stains (Behari and Haldar 1994), these technologies have provided a wealth of information on the formation and function of the exomembrane system.

**Figure 1.**
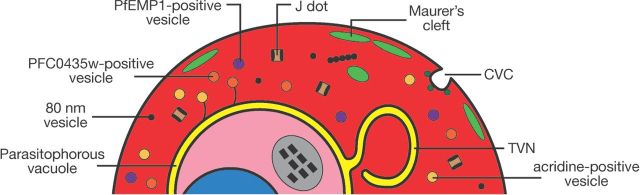
Overview of the elements of the exomembrane system that have been described in the literature. Not all parts have been described in all *Plasmodium* species, and some parts are species-specific. CVC- caveola-vesicle complex; TVN - tubovesicular network

Ultimately, the changes in adhesiveness and permeability exhibited by the infected erythrocyte depend on specific features of the exomembrane system, and all the parasite proteins involved in these changes are in one way or another transferred to the host cell through parts of the exomembrane system. Here we describe the different parts of this exomembrane system (Fig. 1). For the purposes of this review, this is defined as the collection of membranous compartments within the infected erythrocyte beyond the plasma membrane of the parasite that are not present in uninfected erythrocytes. Included are the PV, the tubovesicular network (TVN), the MCs, the caveola-vesicle complex (CVC), J dots and other mobile compartments, and small vesicles. As most experiments have been performed on *P. falciparum*, most of this review will by necessity focus on the exomembrane system of this parasite species, but where differences with other parasites are known, these will also be described.

## THE PARASITOPHOROUS VACUOLE AND PARASITOPHOROUS VACUOLE MEMBRANE

Invasion of erythrocytes by *P. falciparum* is an active and rapid process; in under 60 seconds, extracellular merozoites bind and penetrate host erythrocytes (Dvorak *et al*. 1975; Cowman and Crabb 2006). Subsequently, the intracellular parasite resides in a sealed compartment inside the erythrocyte, distinct from the erythrocyte cytosol—the PV. The PVM that demarcates the PV is a continuous membrane separating the parasite from the erythrocyte cytosol. The lumen of the PV is a small space, with electron micrograph images indicating its diameter to be around 50 nm (Trelka *et al*. 2000). In contrast to the related Apicomplexan parasites of the genus *Babesia*, where the PVM disintegrates shortly after completion of invasion (Rudzinska *et al*. 1976; Repnik *et al*. 2015), *Plasmodium* parasites remain within the PV throughout the intraerythrocytic cycle. As the parasite grows and replicates, the PVM expands to accommodate the growing parasite, but at all stages closely surrounds the parasite. The PVM only ruptures in the final stages of egress of invasive daughter merozoites from the erythrocyte (Blackman and Carruthers 2013).

The PV was first described in the related Apicomplexan parasites *Eimeria perforans, E. stidae* and *Toxoplasma gondii* (Scholtyseck and Piekarski 1965) before being described in *P. falciparum* by Ladda, Arnold and Martin (1966). In electron micrographs, the PV lumen appears substantially less electron dense than the surrounding erythrocyte cytosol (for early examples, see Rudzinska, Trager and Bray 1965; Aikawa 1971), indicating that hemoglobin is unable to cross the PVM. While it is commonplace in electron micrographs to see a distinct space between the parasite and PVM, this has been suggested to be an artifact of chemical fixation (Hanssen *et al*. 2013). Indeed, the PV lumen is not detected when the fixation protocol is switched from glutaraldehyde-based to high-pressure fixation and freeze substitution (Hanssen *et al*. 2013). It was suggested early on that the diameter of the PV in EM images is sensitive to the osmolarity of the fixative (Blackburn and Vinijchaikul 1970).

### Formation of the PV and PVM

The PVM forms very rapidly—in a fraction of a minute (Joiner 1991)—but precisely how the PV and its membrane form remains undetermined. Using an internal membrane system (consisting of the inner membrane complex, actin and surface receptors) attached to the erythrocyte surface through a moving junction, the parasite pushes itself into the cell during invasion of the host erythrocyte (Besteiro, Dubremetz and Lebrun 2011; Harvey *et al*. 2014). Often, the only point of direct contact detected between parasite and erythrocyte membrane is at this junction (for examples, see Ladda, Aikawa and Sprinz 1969; Bannister, Butcher and Mitchell 1977; Aikawa *et al*. 1978; Bannister *et al*. 1986). Although this may again be an artifact of fixation, it may also indicate that the erythrocyte membrane indents without direct contact with the parasite surface.

There is a long-standing controversy over whether the PVM is derived from parasite or host material. The total volume of the PV is miniscule compared to that of the host erythrocyte (less than 1/10 000th the size; Burghaus and Lingelbach 2001) and the small size of the PV presents technical difficulties for specific isolation of the contents of the PV for proteomic analyses. Furthermore, the inability to separate the PVM from other membranes in the infected cell hinders lipidomic analyses. For these reasons, various alternative techniques have been used in an attempt to decipher the origin of the PVM.

**Figure 2.**
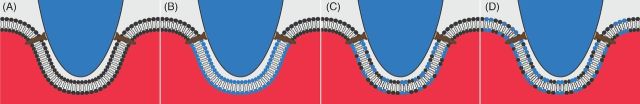
Four potential scenarios to explain the formation of the PVM during invasion of host erythrocytes. The PVM may be derived solely from the host membrane (**A**) or the parasite (**B**). Alternatively, host and parasite components may both contribute directly to the forming PVM (**C**). As a fourth possibility, the erythrocyte may donate membrane to form the PVM with parasite lipids acting to replenish this translocation of host cell membrane (**D**). Host phospholipids are indicated in black, parasite-derived phospholipids are indicated in blue.

Electron micrographs show that, during its formation, the PVM is continuous with the erythrocyte membrane (Aikawa *et al*. 1978). Using fluorescence video microscopy to track fluorescent lipophilic probes used to label the erythrocyte membrane, Ward, Miller and Dvorak (1993) showed that the concentration of such probes in the PVM was indistinguishable from that in the erythrocyte membrane and did not change in concentration during invasion. This led to the conclusion that the PVM is derived from lipids of the erythrocyte membrane with no addition of lipids from exogenous sources (Fig. 2A). A similar conclusion was drawn by Haldar and Uyetake (1992), who used fluorescent lipophilic probes to label the host cell, showing that erythrocyte lipids were internalized upon invasion and ended up in the PVM. However, in both studies the fluorescent probes used were transferred to the intracellular parasite itself over time, indicating that such probes can transfer across aqueous space. Thus, the behavior of such probes may not accurately mirror that of endogenous erythrocyte phospholipids. Pouvelle and colleagues allowed parasites to invade erythrocytes labeled with 1,1-dihexadecyl-tetramethylindocarbocyanine perchlorate (DiIC_16_, a fluorescent, non-exchangeable lipophilic molecule) and observed that fluorescence surrounded the parasite in its vacuole. When DilC_16_-labeled parasites were allowed to invade unlabeled erythrocytes, the fluorescence signal remained within the parasite (Pouvelle, Gormley and Taraschi 1994). This provided further support for the hypothesis that the PVM is exclusively or predominantly host cell derived.

In contrast, other authors have reported that erythrocyte membrane lipids make only a minimal contribution to the PVM, with parasite-derived lipids comprising the majority of the PVM (Fig. 2B). When Mikkelsen and colleagues allowed parasites that had been metabolically labeled with fluorescent lipid precursors to invade erythrocytes, the label initially localized apically in the parasite. However, during invasion the label appeared to be secreted into the erythrocyte membrane from the invading merozoite and was eventually seen in the PVM, surrounding the parasite (Mikkelsen *et al*. 1988). It was concluded that the PVM results from parasite-derived lipids that are inserted into the host cell membrane. In concordance with this, Dluzewski *et al*. (1992) detected much lower levels of chemically labeled host phosphatidylethanolamine in the PVM than in the host cell membrane. This led to the conclusion that there must be a non-erythrocyte membrane source for PVM lipids. An important footnote is that when Ward, Miller and Dvorak (1993) replicated these experiments by tracking fluorescently labeled phosphatidylethanolamine and phosphatidylserine, they detected the labeled phospholipids in the PVM, leading them to derive a different conclusion. Therefore, the debate as to whether the PVM is derived from the erythrocyte or parasite persisted.

Subsequently, Dluzewski and colleagues performed very elegant experiments measuring the change in surface area of the erythrocyte upon parasite invasion. This study showed that there was no appreciable decrease in erythrocyte surface area between uninfected erythrocytes and erythrocytes that had been invaded by one, two, three or even four parasites (Dluzewski *et al*. 1995), leading to the conclusion that the PVM must be derived of lipids from a source other than the erythrocyte (Fig. 2C) or that the erythrocyte membrane used for PVM formation is replenished by parasite-derived lipids (Fig. 2D).

If the PVM is produced, at least in part, from parasite-derived phospholipids, questions over the source of these phospholipids arise. The most likely source is the rhoptries—club-shaped apical secretory organelles unique to Apicomplexan parasites that are released during invasion. Ladda, Aikawa and Sprinz (1969) first posited a role for rhoptries in parasite invasion. The rhoptries decrease in size by over 75% upon invasion (Hanssen *et al*. 2013). Rhoptries in *P. falciparum* and *P. knowlesi* parasites contain lamellar membranous whorls (Bannister *et al*. 1986; Stewart, Schulman and Vanderberg 1986). On the basis of their analogous appearance to lamellar bodies within pulmonary alveolar cells (Joiner 1991), these multilamellar whorls in the rhoptries most likely represent preformed membranes. It has been calculated that rhoptries contain enough membranous material to create a vacuole sufficiently large to encapsulate an invading merozoite (Bannister and Mitchell 1989). During invasion, rhoptry secretion occurs concomitantly with PVM formation (Aikawa *et al*. 1981; Bannister and Mitchell 1989). Indeed, in the presence of cytochalasin D or PMSF, compounds that permit merozoite attachment and rhoptry release while blocking invasion, electron-lucent, hemoglobin-deficient vacuoles are seen within the erythrocyte (Aikawa *et al*. 1981; Dluzewski *et al*. 1989; Riglar *et al*. 2011). These vesicles receive lamellar material from the parasite, indicating transfer of parasite phospholipid from the parasite into a vacuole-like structure (Dluzewski *et al*. 1989). Therefore, the parasite rhoptries possess the capacity to be a major contributor of lipids during PVM formation. However, not all rhoptry membrane is deposited in the PVM. In *P. knowlesi* and *P. falciparum*, some rhoptry membrane persists in the merozoite once the PVM has fully formed (Bannister *et al*. 1975; Hanssen *et al*. 2013) and this may have a structural role within the merozoite itself.

PVM composition may provide clues to its biogenesis. Freeze-fracture (McLaren *et al*. 1977) and immuno-EM (Atkinson *et al*. 1988; Dluzewski *et al*. 1989), as well as fluorescence microscopy (Ward, Miller and Dvorak 1993), have shown that the PVM surrounding *P. falciparum* and *P. knowlesi* parasites lacks all major erythrocyte membrane and cytoskeletal proteins, including spectrin, ankyrin and band 3. It has been suggested that this is evidence that the PVM is not erythrocyte derived (Atkinson *et al*. 1988), although such findings do not automatically preclude the erythrocyte from contributing to the PVM (Dluzewski *et al*. 1989). During invasion by *T. gondii*, the tight junction acts as a molecular sieve (Mordue *et al*. 1999) and it is feasible that a similar exclusion process in *Plasmodium*-infected erythrocytes allows phospholipids, but not proteins, to pass. Interestingly, in the related Apicomplexan *Babesia divergens*, glycophorin A, spectrin and band 3 are present in the PVM (Repnik *et al*. 2015). Repnik and colleagues hypothesize that such erythrocyte membrane proteins are also present in the *P. falciparum* PVM, but are rapidly degraded by parasite proteases.

Although the PVM is devoid of most major erythrocyte proteins, several authors have detected some (but not all) proteins that reside in cholesterol-rich detergent-resistant membrane rafts (DRMs) in the PVM (Lauer *et al*. 2000; Nagao, Seydel and Dvorak 2002; Murphy *et al*. 2004). This led to the hypothesis that such erythrocyte-derived lipid rafts contribute to the PVM. Low-level removal of cholesterol from the erythrocyte does not affect erythrocyte membrane function but does block DRM formation, prevent DRMs forming vacuoles and block invasion by the parasite (Samuel *et al*. 2001). Therefore, it is possible that, during invasion, the parasite recruits DRMs from the erythrocyte membrane, and this aids in PVM formation.

As well as rhoptry lipids potentially playing an important role in PVM formation, proteins contained within the rhoptries may also be involved (Riglar *et al*. 2011). As shown by fluorescence microscopy and immuno-EM, secretion of rhoptry proteins occurs concurrently with PVM formation (Bannister *et al*. 1986; Carruthers and Sibley 1997; Riglar *et al*. 2011). For example, a *Plasmodium* spp*.* ortholog of the erythrocyte cytoskeletal protein stomatin, Pfstomatin, is hypothesized to recruit DRMs from the erythrocyte membrane to form the PVM (Hiller *et al*. 2003). Parasite and host proteins may play key roles in active recruitment of DRMs to the PVM. However, it is also possible that the physical forces generated by the parasite forming its initial invasion pit may act to destabilize the erythrocyte membrane (Bannister *et al*. 1975) thereby triggering coalescence of DRMs into a vacuole-like structure (Murphy *et al*. 2007).

In conclusion, several decades of elegant and complicated experiments have done little to settle the question of whether the PVM is parasite or host derived (Topolska *et al*. 2004; Bietz *et al*. 2009; Zuccala and Baum 2011). However, it seems most likely that the erythrocytic contribution is likely one of initial membrane material: this is then extensively added to and altered by parasite material derived from the rhoptries.

### Expanding the PVM

As the intracellular parasite grows, the PVM expands to accommodate it, with the width of the lumen of the PV, the space between the PVM and the parasite plasma membrane, remaining roughly constant at ∼20–30 nm (200–300 Å) throughout the intraerythrocytic life cycle (Ladda, Arnold and Martin 1966) (although as mentioned above, there is debate about how accurately the width of the PV is represented in EM images owing to the potential for artifacts of the fixation procedure). How the expansion of the PV is regulated so that it expands at a similar rate as the parasite and the origin of the phospholipids that allow the expansion are unknown. As the erythrocyte is devoid of internal membranes and has at most a severely reduced capacity to produce phospholipids (Marks, Gellhorn and Kidson 1960), the host cell cannot be the source of the membranes. The parasite makes a full suite of phospholipids (Dechamps *et al*. 2010) and during *in vitro* culture does not rely on exogenous phospholipids (although it does require fatty acids from the medium; Asahi *et al*. 2005; Asahi 2009), indicating that the source of the phospholipids for the expansion of the PVM must be the parasite. However, there is no obvious mechanism for the transport of parasite-derived phospholipids through the aqueous lumen of the PV to the PVM. Similarly, there is no indication that there is a direct physical connection between the PVM and the erythrocyte membrane or that there is vesicular transport between these two membranes that may deliver phospholipids to the PVM. The parasite produces a broad-specificity phospholipid transport protein, PFA0210c, which is exported to the erythrocytes and also resides in the PV (van Ooij *et al*. 2008, 2013). As PFA0210c can transfer phospholipids between membranes, it is possible that this protein may accept phospholipids in the parasite (either the ER or the parasite plasma membrane) and deliver these to the PVM. It is not clear how the directionality or rate of this phospholipid transfer might be regulated. Furthermore, how the shape of the PVM and relatively stable distance between the parasite plasma membrane and the PVM are maintained remain to be discovered.

### Necklace of beads

One curious aspect of the PV is a compartmentalization that occurs early in the intraerythrocytic stages. Transgenically expressed fluorescent proteins that are secreted into the PV are detected in a dot-like pattern, referred to as a ‘necklace of beads’, instead of the continuous circular pattern that would be expected if the PV were one uninterrupted compartment. This pattern is detected primarily in young rings (Wickham *et al*. 2001; Adisa *et al*. 2003; Spielmann *et al*. 2006); at later stages, the PV does form a continuous compartment, as judged by the circular localization of soluble fluorescent proteins. Investigations using fluorescence recovery after photobleaching (FRAP) experiments have revealed that the diffusion of the soluble fluorescent proteins between the compartments is slower than expected for freely diffusing proteins (Wickham *et al*. 2001; Adisa *et al*. 2003), indicating that while there is a connection between the ‘beads’, it is likely constricted, preventing free diffusion of the proteins. The export apparatus that transfers proteins across the PVM (described in more detail below) is also detected in this ‘necklace of beads’ pattern in early rings (Bullen *et al*. 2012; Riglar *et al*. 2013), although it is unclear whether this affects its function, or reflects the organization of PV and the PVM. Exactly how beads are formed and if they have a specific role or are merely a remnant of the mechanism of PV formation remains to be determined.

### Transport across the PVM

The PVM forms a significant barrier to the uptake of nutrients and the export of proteins. Nutrients pass the erythrocyte membrane through nutrient transporters and then need to traverse the PVM to reach the parasite. Moving in the opposite direction are the proteins exported by the parasite to modify many of the properties of the erythrocyte; these need to be translocated from the PV lumen to the erythrocyte cytosol. Both processes are essential for the parasite (Divo *et al*. 1985; Asahi *et al*. 2005; Asahi 2009; Beck *et al*. 2014; Elsworth *et al*. 2014) and interestingly, they may involve the function of one parasite protein, namely EXP2.

Elegant patch clamp experiments on parasites freed from the erythrocyte revealed the presence of a pore in the PVM that allows the passive diffusion of solutes up ∼1400 Da (Desai, Krogstad and McCleskey 1993; Desai and Rosenberg 1997). Under the conditions tested, this pore was open 98% of the time (Desai and Rosenberg 1997), allowing for nearly uninterrupted transport of nutrients. The PVM of the related apicomplexans *T. gondii* (Schwab, Beckers and Joiner 1994) and *E. nieschulzi* (Werner-Meier and Entzeroth 1997) also allows diffusion of solutes, with a very similar size cut-off. Two *T. gondii* proteins, GRA17 and GRA23, were recently identified as forming the solute pore (Gold *et al*. 2015). GRA17 appears to be the major pore component, as *GRA17* null mutants have a much more severe phenotype than *GRA23* null mutants; a double mutant could not be obtained, hence GRA23 must partially complement the function of GRA17. Interestingly, overexpression of *GRA17* increased the growth rate of the parasites, indicating that nutrient uptake may be the limiting factor in the growth of *T. gondii*. The PV of *GRA17* mutant parasites becomes expanded compared to wild-type parasites and often collapses, and its membrane is impermeable to small fluorescent solutes that readily diffuse into the PV of wild-type parasites. Furthermore, GRA17 and GRA23 changed the conductivity of *Xenopus laevis* oocytes injected with mRNA encoding these proteins, showing directly that these proteins can function as pores. The functional ortholog of GRA17 in *Plasmodium* spp. is EXP2. EXP2 was initially discovered as a PVM protein that is abundantly expressed throughout the entire intraerythrocytic lifecycle (Johnson *et al*. 1994; Fischer *et al*. 1998), and hence is in the expected subcellular location to function as a nutrient channel. Complementation of a *T. gondii GRA17* mutant with *P. falciparum EXP2* reinstated the wild-type phenotype, showing the functional equivalence of the two genes. GRA17, GRA23 and EXP2 have distant similarity to hemolysin E of *Escherichia coli*, a protein that multimerizes within membranes to form pores, further supporting their role as a pore and suggesting a mechanism through which the proteins are inserted into the membrane (Parker and Feil 2005).

In contrast to nutrient uptake, transport of proteins from the PV to the erythrocyte cytosol is mediated by a large protein complex, termed the *Plasmodium* translocon of exported proteins (PTEX) (de Koning-Ward *et al*. 2009; Bullen *et al*. 2012; Riglar *et al*. 2013; Beck *et al*. 2014; Elsworth *et al*. 2014; Spillman, Beck and Goldberg 2015). This complex was initially thought to consist of at least five proteins: PTEX150 and PTEX88, two proteins with unknown functions; the ATPase HSP101, which is thought to supply the energy for translocation (El Bakkouri *et al*. 2010); the thioredoxin Trx2; and EXP2, which has been speculated to form the central translocation channel, also based on its distant resemblance to *E. coli* hemolysin E (de Koning-Ward *et al*. 2009). The finding that some improperly folded exported proteins jam the export channel and that binding of skeleton binding protein 1 (SBP1) to EXP2 is more readily detected when it is improperly folded has further provided evidence that EXP2 could indeed be the portal through which exported proteins cross the PVM (Mesen-Ramirez *et al*. 2016). Several additional proteins that appear to associate with the PTEX, including PfPV1, Pf113 and Hsp70-x, have since been discovered, although the association of these protein with the PTEX may be weaker and more transient than that of the five core components (Elsworth *et al*. 2016; Mesen-Ramirez *et al*. 2016). Based on solubility experiments, EXP2 appears to be the membrane-bound component of the PTEX (Bullen *et al*. 2012), whereas the other components are peripherally associated with the membrane and likely are involved in recruiting client proteins to the EXP2 pore. The predicted size of the pore formed by hemolysin E is large enough to accommodate polypeptide chains (Parker and Feil 2005), indicating that unfolded proteins may be able to pass through a pore formed by EXP2. Interestingly, although protein export is an essential process (Beck *et al*. 2014; Elsworth *et al*. 2014), mutants lacking Trx2 or PTEX88 have been obtained in *P. berghei* (Matthews *et al*. 2013; Matz *et al*. 2015b) (although one group has reported that PTEX88 is refractory to deletion (Matthews *et al*. 2013), which may reflect the difference in the selection of the mutants (FACS and drug) in the two studies). The thioredoxin mutant has a growth defect *in vivo* and *in vitro* (Matthews *et al*. 2013), whereas the PTEX88 mutant exports proteins with an efficiency indistinguishable from wild-type parasites, but loses the ability to sequester and grows slower (Matz *et al*. 2015b; Chisholm *et al*. 2016). Such a specific phenotype of a mutant of the export machinery may indicate that individual components of the translocon may be required to direct the export of only a subset of exported proteins.

Perhaps surprisingly, existing data suggest that the location of the PTEX varies according to the species and the host cell. The *P. falciparum* PTEX is located in the PVM (Bullen *et al*. 2012; Riglar *et al*. 2013), but in *P. berghei* the complex is also present in loops resembling the TVN (Matz *et al*. 2015a; Meibalan *et al*. 2015). Similarly, when growing in reticulocytes, the *P. falciparum* PTEX complex, as indicated by the presence of EXP2, is also partially located outside of the PVM (Meibalan *et al*. 2015).

Thus, one protein, EXP2, appears to be involved in both processes, the transport of solutes and proteins across the PVM, as was initially suggested by Gold *et al*. (2015). One interesting feature of the PTEX is that the expression profiles of the individual components are different; *EXP2* is transcribed almost uniformly throughout the intraerythrocytic life cycle, whereas transcription of the other four components peaks late in the life cycle, as often seen with proteins that are packaged in the merozoite and released after invasion (Bullen *et al*. 2012). As the components of large macromolecular complexes are often synthesized at the same time, this may further indicate potential alternate functions for the individual parts of the PTEX. EXP2 is produced during the liver stage of infection (this stage precedes the erythrocytic cycle in the parasite lifecycle), as are PTEX components PTEX150, PTEX88 and Trx2 (Vaughan *et al*. 2012; Matz *et al*. 2015b; Kalanon *et al*. 2016), whereas HSP101 is not detected at this stage (Matz *et al*. 2015b; Kalanon *et al*. 2016). Furthermore, the parasite load in the livers of mice infected with *EXP2* null parasites was significantly decreased compared to the parasite load in mice infected with control parasites (Kalanon *et al*. 2016). Previously, only two proteins have been reported to be exported during the liver stage (Singh *et al*. 2007; Orito *et al*. 2013), although a well-established reporter for protein export is not exported (Kalanon *et al*. 2016). Hence, during the liver stage, in the absence of the other PTEX components, EXP2 may function solely as a nutrient transporter. Possibly, this may reflect the origin of EXP2, which in the ancestor of the Apicomplexans may have functioned solely as a solute pore, but during subsequent speciation have been co-opted to function in parallel as a protein pore through recruitment of additional proteins that were already present in the parasite (HSP101 and Trx2) and possibly newly evolved (PTEX150 and PTEX88). Interestingly, Trx2 has also been detected in an unidentified internal compartment (Kehr *et al*. 2010), perhaps indicating that the original function of Trx2 is in this organelle and that it was recruited for protein export only later.

### Formation of the cytostome and hemoglobin uptake

In addition to uptake of nutrients from the surroundings, degradation of hemoglobin inside the parasite is an important source of nutrients for the parasite and increases the space available within the erythrocyte to accommodate the growing parasite, although viable *P. berghei* parasites that neither digest hemoglobin nor produce hemozoin have been produced (Lin *et al*. 2015). Hemoglobin degradation in the parasite takes place within the digestive vacuole (also referred to as the food vacuole), a lysosome-like organelle containing enzymes that break down hemoglobin and convert heme, a toxic catabolite, into non-toxic hemozoin (Goldberg 2005). As with the uptake of nutrients, the PVM forms a barrier to the uptake of hemoglobin, which in this case is overcome by a specialized structure in the PVM called the cytostome (previously referred to as the ‘micropyle’) (Garnham *et al*. 1961; Rudzinska, Trager and Bray 1965; Aikawa, Huff and Spinz 1966; Aikawa 1971; Seed *et al*. 1976). This appears as a cup-shaped, double-membraned invagination of the PVM and the parasite plasma membrane, the lumen of which is continuous with the erythrocyte cytosol (Yayon *et al*. 1984); EM images clearly reveal the presence of electron-dense hemoglobin in this compartment (Aikawa, Huff and Spinz 1966; Yayon *et al*. 1984). The neck portion of the invagination, where it connects with the parasite plasma membrane and the PVM, is approximately 50 nm wide and is surrounded by an electron dense ring (Langreth *et al*. 1978), which was recently shown to be a double-layered ring (Milani, Schneider and Taraschi 2015) that contains actin. Experiments using erythrocytes loaded with fluorescent beads of various sizes showed that particles up to 50–70 nm in diameter could be taken up by the cytostome (Goodyer *et al*. 1997). It was originally thought that the cytostome pinches off from the parasite plasma membrane and the PVM to form a double-membraned vesicle that transports hemoglobin to the digestive vacuole (Yayon *et al*. 1984), but more recent investigations support the model that the cytostome is a static structure in the parasite from which vesicles bud off to deliver hemoglobin (Milani, Schneider and Taraschi 2015), a process that likely involves Rab5 (Elliott *et al*. 2008; Ezougou *et al*. 2014).

Cytostomes form soon after invasion, and can also be detected on merozoites (Bannister and Mitchell 2009; Hanssen, McMillan and Tilley 2010), and remain present throughout the intraerythrocytic cycle (Lazarus, Schneider and Taraschi 2008). Each intracellular parasite contains on average two to three cytostomes (Milani, Schneider and Taraschi 2015), but parasites with more cytostomes have been detected (Aikawa and Jordan 1968; Slomianny 1990; Abu Bakar *et al*. 2010; Milani, Schneider and Taraschi 2015); usually these multiple cytostomes are in close proximity. Therefore, it appears that cytostomes in the intracellular parasites either originate from an invagination that already exists in the merozoite or are newly formed cytostomes. Recent studies of the role of iron in artemisinin resistance in *P. falciparum* and electron tomography studies using *P. chabaudi*-infected erythrocytes have indicated that hemoglobin breakdown initiates early in the ring stage, indicating that the cytostomes seen in rings are actively taking up hemoglobin (Wendt *et al*. 2016; Xie *et al*. 2016).

The function of cytostomes depends on dynamin, an ATPase that promotes the pinching off of clathrin-coated vesicles. Within parasites, the dynamin Dyn1 localizes to small regions near the plasma membrane (Li *et al*. 2004; Zhou *et al*. 2009). The dynamin inhibitor dynasore blocks the transport and breakdown of hemoglobin (Zhou *et al*. 2009; Milani, Schneider and Taraschi 2015) and alters the shape of the cytostome, but does not prevent cytostome formation (Milani, Schneider and Taraschi 2015). Actin polymerization likely plays an important role in the formation or stabilization of the cytostome, as treatment with jasplakinolide or cytochalasin D causes elongation of the cytostome, accumulation of electron-dense, hemoglobin-containing vesicles in the parasite and a slight decrease in the number of cytostomes (Milani, Schneider and Taraschi 2015).

How the cytostome is formed remains unclear—a double-membraned invagination is an uncommon structure so there are few, if any, examples that could provide insight into its formation. Formation of cytostomes requires the membranes of the PVM and parasite plasma membrane to come together and invaginate, a process that undoubtedly relies on the function of dedicated (unidentified) parasite proteins in the parasite plasma membrane and the PVM. It has been speculated that the cytostome is produced during the ‘big gulp’, a movement of the parasite during which it changes from a relatively flat, cup-shaped cell to a spherical cell, where it is left at the far end of the parasite (Elliott *et al*. 2008). However, this model cannot explain the presence of multiple cytostomes in the parasite, and hence how the cytostome is produced remains unknown.

The role of cytostomes in the transport of digestive enzymes to the digestive vacuole is similarly unclear; several mechanisms have been proposed, including transport of proteins through the PV, leading to entrapment of the enzymes between the membranes of the cytostome and subsequent transport to the digestive vacuole (Francis *et al*. 1994), and direct transport of the proteins to the cytostome from the secretory organelles (Klemba *et al*. 2004). The lack of marker proteins hampers its further investigation, but the cytostome is most likely of great importance to the parasite and further study of this compartment will very likely reveal very interesting new biological principles.

Potentially related to the uptake of hemoglobin is the central cavity (also referred to as ‘spherical structure’) that has been described in *P. falciparum* and *P. berghei* (Abu Bakar *et al*. 2010; Grüring *et al*. 2011). This is a large indentation in the surface of the parasite that extends into the center of the parasite. It is filled with erythrocyte cytosol and excludes GFP present in the parasite cytosol. This compartment appears similar to a large hemoglobin-containing compartment that has been detected in various EM studies (Rudzinska, Trager and Bray 1965; Trager, Rudzinska and Bradbury 1966; Yayon *et al*. 1984). Although the central cavity appears internal to the parasite, it is directly connected to the erythrocyte cytosol. The central cavity develops in the ring stage and persists until the parasite matures into a schizont. Digestive vacuoles are detected in close proximity to the central cavity, and although the functional significance of that is not understood it does indicate that these compartments are separate entities (Grüring *et al*. 2011).

### The PV and PVM during egress

At the end of the erythrocytic cycle, merozoites are released by the rupture of the PVM and the erythrocyte membrane. It is believed that the PVM lyses before the erythrocyte (Bosia *et al*. 1993; Wickham, Culvenor and Cowman 2003; Blackman and Carruthers 2013), despite an earlier report that the erythrocyte membrane lyses first (Salmon, Oksman and Goldberg 2001). Immediately prior to egress, the parasite releases the protease SUB1, and likely other proteins, into the PV through the release of the contents of the exonemes in a protein kinase G-regulated process (Yeoh *et al*. 2007; Collins *et al*. 2013b). In the PV, SUB1 cleaves several proteins, including members of the MSP1 complex (Child *et al*. 2010; Das *et al*. 2015), SERA5 and SERA6 (Yeoh *et al*. 2007; Ruecker *et al*. 2012) and potentially other proteins (Silmon de Monerri *et al*. 2011), but what causes the dissolution of the PVM is not clear. As the membrane is permeable to solutes, its rupture is unlikely to be mediated by changes in osmolarity and may require specific proteins. Perforin-like protein 1 (PLP1), a member of the membrane-attack complex/perforin superfamily of proteins that can disrupt phospholipid bilayers by forming transmembrane pores, is required for the release of *T. gondii* from the host cell (Kafsack *et al*. 2009; Roiko and Carruthers 2013). In host cells infected with a mutant lacking TgPLP1, the PVM remains intact when egress is induced with an ionophore and the mutant is avirulent *in vivo* (Kafsack *et al*. 2009). *Plasmodium* spp. encode five PLPs (Carlton *et al*. 2002; Gardner *et al*. 2002), of which two are expressed during the blood stages (Kaiser *et al*. 2004; Garg *et al*. 2013). However, *P. berghei* mutants lacking either one of the genes encoding these proteins, PbPPLP1 and PbPPLP2, do not display an obvious phenotype in the asexual lifecycle, although gametocytes (sexual blood-stage forms that develop from the asexual stages) lacking PbPPLP2 are unable to egress and are transmitted to the mosquito vector inefficiently (Ishino, Chinzei and Yuda 2005; Deligianni *et al*. 2013). Investigation of PPLP2 in *P. falciparum* revealed that it is selectively required for the breakdown of the erythrocyte membrane in gametocytes; *PfPPLP2* null mutants undergo PVM rupture similarly to wild-type parasites (Wirth *et al*. 2014). In asexual blood stages, PfPPLP1 is secreted into the PV and is localized next to the PVM and the erythrocyte plasma membrane (Garg *et al*. 2013). Although the effect of deletion of this protein is not known, it may be responsible for PVM rupture and/or the ‘poration’ of the erythrocyte membrane that occurs shortly before its rupture (Glushakova *et al*. 2010). On the other hand, PVM rupture in liver stages of *P. berghei* clearly involves a role for a parasite phospholipase (Burda *et al*. 2015), raising the possibility that a similar enzyme may be involved in blood-stage egress. Hence, the mechanism of breakdown of the PVM is unclear and needs to be investigated further.

### Roles of the PV and PVM

Several roles for the PV and PVM have been proposed (Lingelbach and Joiner 1998), but the exact functions of the PV and the PVM remain unclear. As the PVM forms such a large barrier and the parasite has the ability to break down the PVM, as seen during egress, it is unlikely that the PV is simply a remnant of the invasion process and therefore likely performs an important function(s). Several related apicomplexans, including *Theileria* spp. and *Babesia* spp. (Rudzinska *et al*. 1976; Fawcett, Musoke and Voigt 1984; Asada *et al*. 2012; Repnik *et al*. 2015), exit the PV soon after entry into the host cell and subsequently reside free in the cytosol. Therefore, the erythrocyte cytosol does not appear to be an inhospitable environment for parasite growth.

Limiting our understanding of the roles of the PV and PVM in *Plasmodium* spp. is the low number of PV and PVM proteins that have been functionally characterized (Spielmann *et al*. 2012). Using a biotinylation approach that specifically modified soluble PV proteins, a proteomics study identified 27 proteins present in the PV at the late ring/early trophozoites, including the known PV proteins glycophorin-binding protein 130 and SERP (SERA5) (Nyalwidhe and Lingelbach 2006). All members of the family of SERA proteins contain a signal sequence and a papain-like domain and those that are expressed during the intraerythrocytic stage may be present in the PV. Whereas some are likely to be active proteases, the active site cysteine has been replaced with a serine in SERA5 and several other members of the family, rendering them enzymatically non-active (Arisue *et al*. 2011; Stallmach *et al*. 2015). Interestingly, over half of the identified PV proteins were proteases or chaperones, indicating that protein folding and processing is likely to be an important aspect of the PV. In addition, merozoite surface proteins MSP7 and members of the MSP3 family were identified, although this may reflect a step in the formation of protein complexes on the merozoite surface rather than a function of these proteins in the PV itself. This study identified PfPV1, which was later shown to be an essential PV protein (Chu, Lingelbach and Przyborski 2011) and found to associate with the PTEX (Elsworth *et al*. 2016), although its function remains unknown. Other known soluble PV proteins include the merozoite surface proteins S-antigen (Culvenor and Crewther 1990), GLURP (Borre *et al*. 1991) (which are encoded in the same genetic region as the MSP3 family members) and ABRA (MSP9) (Stahl *et al*. 1986; Chulay *et al*. 1987), and the proteases SERA6, an active cysteine protease (Ruecker *et al*. 2012), and SUB1, a subtilisin-like protease. Whereas all other PV proteins listed here are transported to the PV through the secretory pathway, SUB1 is released from exonemes, which are specialized secretory organelles that release their contents into the PV in a protein kinase G-dependent manner at a very late stage in the intraerythrocytic cycle (Yeoh *et al*. 2007; Collins *et al*. 2013b).

EXP1, EXP2 and members of the ETRAMP family are the only well-established PVM proteins (Simmons *et al*. 1987; Johnson *et al*. 1994; Spielmann, Fergusen and Beck 2003; Spielmann *et al*. 2006), and of these, only EXP2 can be assigned a function, as described above, although EXP1 may function as a glutathione S transferase (Lisewski *et al*. 2014).

The large fraction of PV proteins that function in the processing of other proteins, either through proteolysis or protein folding, may reflect the need for exported proteins to be unfolded prior to export (Gehde *et al*. 2009) and the extensive processing of protein complexes on the merozoite surface. Potentially, the main function of the PVM is to retain SUB1 and other PV enzymes in a small volume to promote the rapid maturation of other soluble PV, PMV and merozoite surface proteins. It is also possible that the cytosol of the erythrocyte is a less-than-hospitable environment from which the parasite needs to be shielded and that *Theileria* spp. and *Babesia* spp. have evolved mechanisms to counteract the damaging effects of this environment. The phenotype of a mutant *Plasmodium* parasite that escapes the PV following invasion would provide great insight into the role of the PV.

## THE TUBOVESICULAR NETWORK

The TVN is the least defined and most poorly understood part of the exomembrane system. For the purpose of this review, it is defined as the membranous compartment that is contiguous with the PVM but that is not positioned adjacent to the parasite plasma membrane. The TVN is most often detected as a loop that protrudes from the PVM into the erythrocyte cytosol. In EM images (for example, see Kara *et al*. 1988; Elford, Cowan and Ferguson 1995, 1997), it can frequently be detected as a nearly complete loop that encircles hemoglobin, although it is not clear from these 2D images whether it forms a complete sphere in which hemoglobin is trapped. EM images of parasites released by osmotic lysis from the erythrocyte often reveal a long, slender protrusion from the PVM, possibly indicating that the loop is not fully folded back on itself (Elford, Cowan and Ferguson 1995). Whereas these experiments indicate that there is a physical connection between the PVM and the TVN membrane, the presence of a continuous lumen is less clear. Soluble PV-targeted GFP in some cases also labels the TVN (Wickham *et al*. 2001; Adisa *et al*. 2003), indicating a direct connection, and FRAP experiments revealed that in some instances the fluorescence signal in the TVN can be recovered although in other cases no recovery was detected, even within the same sample (Wickham *et al*. 2001; Adisa *et al*. 2003). EM images revealed a possible constriction at the junction of the PVM and the TVN (Elford, Cowan and Ferguson 1995). How this relates to the inconsistency in the results of the FRAP experiments is not clear; possibly there are changes in the properties of the TVN and its connection to the PVM as the intracellular parasite matures.

The TVN was first visualized by fluorescence microscopy by Haldar and colleagues using fluorescent ceramide analogs (Behari and Haldar 1994; Lauer *et al*. 1997). The TVN appears to contain high levels of sphingolipids, as this organelle is brightly stained after the addition of fluorescent ceramide, and remains stained even after back extraction (Haldar *et al*. 1991), leaving only the fluorescent ceramide that has been converted to sphingomyelin. However, ceramide also readily stains other intraerythrocytic organelles, such as the MCs, and is often used as a general membrane stain.

Inhibitors of sphingomyelin synthesis induce dissipation of the TVN and a decrease in the uptake of nutrients (Lauer *et al*. 1997). This has been interpreted to indicate that the TVN plays an important role in nutrient uptake. As the inhibitor treatment was performed over a long period (12 h), it is possible that other processes in the parasite were also affected, independently adding to the decrease in nutrient uptake. Hence, the role of the TVN in the uptake of nutrients requires further investigation.

Study of the TVN has been hampered by the lack of distinct protein markers. EXP1 and EXP2 are present on the TVN (Kara *et al*. 1988; Johnson *et al*. 1994; Lauer *et al*. 1997; Fischer *et al*. 1998), but as these markers also visualize the PVM, their utility as TVN markers is limited. The protein PFC0435w (also known as parasite-infected erythrocyte surface protein (PIESP1)) was initially localized to the surface of the host erythrocyte (Florens *et al*. 2004), but a PFC0435w-GFP fusion was detected at the junction of the PVM and the TVN (van Ooij *et al*. 2008). This protein contains a fringe-like domain characteristic of proteins involved the transfer of glycosyl residues (Yuan *et al*. 1997). Fringe-like proteins are generally secreted and play important signaling roles in development in higher eukaryotes (Takeuchi and Haltiwanger 2010) and the formation of lipooligosaccharide in bacteria (Sirisena *et al*. 1992; Chen and Coleman 1993). PFC0435w may therefore play an important role in signaling in the PVM or in the modification of merozoite surface proteins. The presence of GFP at the C terminus in the fusion protein may obscure a potential ER-retention signal (TDEL) in the C terminus of the protein (Raykhel *et al*. 2007), although the C termini of PFC0435w orthologs in other *Plasmodium* spp. fit the consensus for an ER retention signal much less (the C termini of the *P. knowlesi* and *P. chaubaudi* orthologs are EAGEL and YNTEL, respectively), indicating that the protein may not be retained in the ER. The apparent essentiality of the gene encoding PFC0435w in *P. falciparum* (Maier *et al*. 2008) and *P. berghei* (van Ooij *et al*. 2008) indicates that this protein performs an important function, but this has also prevented the genetic investigation of a potential role in TVN formation or maintenance. Furthermore, the exported protein PFD0495c was reported to have a role in the acquisition of phospholipids for the TVN (Tamez *et al*. 2008), but this appears not to be an essential role as parasites lacking the PFD0495c gene are viable (Maier *et al*. 2008).

The function of the TVN thus remains unresolved, and the lack of specific markers to visualize this organelle provides an obstacle to further study. Potentially it forms a reservoir of lipids for the expanding PVM or it may have a function in sensing the conditions within the erythrocyte. It has also been suggested that it increases the surface area facing the erythrocyte cytosol and thereby increases nutrient uptake (Lingelbach and Joiner 1998). As the TVN is an elaborate structure with a distinct morphology, there must be a dedicated set of proteins that build and maintain this organelle. While challenging, identification of these proteins will allow this important organelle to be investigated in much greater detail.

## MAURER'S CLEFTS

The initial observation of host cell modification by *Plasmodium* parasites was made by George Maurer, who detected staining within infected erythrocytes outside of the parasite (Maurer 1902). These modifications, the MCs, are the most striking and best characterized—molecularly as well as functionally—part of the exomembrane system. The first detailed examination of the MCs was provided by Trager, Rudzinska and Bradbury (1966) in EM images of blood from individuals infected with *P. falciparum*, in which they detected ‘narrow clear clefts bounded on each side by two unit membranes’ in the erythrocyte cytosol. MCs are membranous compartments of 500–600 nm in length. Morphology varies between parasite strains, although in most isolates the MCs are single membranous compartments with a lumen that appears less dense than the erythrocyte cytosol. MCs are normally present underneath the erythrocyte membrane, but, despite their name, are not connected to the extracellular space. Initially they were thought to be cast-off parts of the limiting membrane, but they have since been shown to form a central component of the system that transports parasite proteins to the erythrocyte surface.

### Formation of MCs

A ground-breaking study by Grüring *et al*. (2011) that imaged infected erythrocytes in 3D over the entire intraerythrocytic lifecycle showed that the MCs, contrary to earlier beliefs, are made very early after invasion, from which point on the number of MCs remains constant at around 15 per infected erythrocyte; multiply-infected erythrocytes contain ‘proportionally more clefts’ (Cooke *et al*. 2006). Early MCs contain only some of the markers that are associated with mature MCs (for example REX1, but not SBP1), indicating that there must be transfer of proteins from the parasite to the MCs after the MCs have been formed (Grüring *et al*. 2011). Early speculation that the MCs remain connected to the PVM throughout the intraerythrocytic cycle, based on EM images of serial sections of infected erythrocytes (Wickert *et al*. 2003), was resolved by careful tomography showing that MCs are separate, unconnected compartments (Hanssen *et al*. 2008b) and that little transfer of contents between MCs occurs (Grüring *et al*. 2011).

In parasites that have lost REX1 (Hanssen *et al*. 2008a; Dixon *et al*. 2011; McHugh *et al*. 2015) or the region of chromosome 9 that encodes REX1 through REX 4 (such as in the D10 strain of *P. falciparum*; Day *et al*. 1993) the MCs appear as multiple, stacked discs. Similar stacked MCs are detected in the Saint Lucia strain of *P. falciparum* (Aikawa *et al*. 1986). That REX1 is present in MCs from a very early time point (Grüring *et al*. 2011) may indicate that it plays an important part in formation of the compartments.

### Interaction of MCs with the host cytoskeleton

For the first 20–24 h of the intraerythrocytic lifecycle, the MCs are present as motile compartments, after which they attach to the cytoskeleton and remain immobile (Grüring *et al*. 2011). Several MC resident proteins bind to proteins in the erythrocyte cytoskeleton, including SBP1, which binds the cytoskeletal proteins 4.1R and spectrin (Blisnick *et al*. 2000; Kats *et al*. 2015) and Pf332, which binds actin (Waller *et al*. 2010). In parasites lacking SBP1, MCs are slightly thinner and lie slightly further from the erythrocyte membrane, but there are no gross alterations in their morphology (Cooke *et al*. 2006; Maier *et al*. 2007; Kats *et al*. 2015). Furthermore, SBP1 is already present on the MC when it is still motile (Grüring *et al*. 2011), so additional factors must be required for the attachment to the cytoskeleton. In contrast, in the absence of Pf332 the number of MCs decreases while the size of the MCs increases and they become stacked (Glenister *et al*. 2009).

One protein that may form the link between MCs and the cytoskeleton is MAHRP2. This protein is detected in an electron dense tether of approximately 100–200 nm in length and a width of 30–50 nm that appears to connect the MC to the erythrocyte cytoskeleton or membrane (Pachlatko *et al*. 2010). A similar tether was detected in electron tomography studies of infected erythrocytes (Hanssen *et al*. 2008b). In the initial report describing MAHRP2, the protein was detected from the trophozoite stage onwards, making it tempting to speculate that it is the appearance of MAHRP2 (and potentially other proteins of the tether) on the MC that tethers the MC to the cytoskeleton, thereby allowing the initiation of protein transport to the surface. However, a more recent report has shown that MAHRP2 is already present in young rings and colocalizes with the MC marker REX1, although in the early stages, not all the MCs that contain REX1 also contains MAHRP2 (McMillan *et al*. 2013). The inability to disrupt the gene encoding MAHRP2 has hampered further investigations, but does indicate the important role of this protein (Pachlatko *et al*. 2010). In addition, deletion of the gene encoding PfPTP1, an MC-resident protein that interacts with several other MC resident protein, affects the actin network in the host erythrocyte and also leads to a severe change in the morphology of the MCs, although the mutant parasites are viable. Potentially PfPTP1 interacts with the cytoskeleton directly or is required for the proper of transport of a protein (or proteins) that mediates the attachment of the MCs to the actin cytoskeleton (Rug *et al*. 2014).

### Sorting of proteins to the erythrocyte surface

The function of the MCs is thought to be the sorting of parasite proteins to the erythrocyte surface. The first evidence for this came when the loss of electron dense material surrounding the MCs was noted in knob-less parasites, and a similar loss of electron dense material was seen at the schizont stage, when the formation of knobs ends (Aikawa *et al*. 1986). Many parasite proteins known to be transported to the surface or the cytoskeleton of the infected erythrocyte are detected at the MCs, including KAHRP (Wickham *et al*. 2001), PfEMP1 (Kriek *et al*. 2003; Wickert *et al*. 2003) and members of the STEVOR (Kaviratne *et al*. 2002; McRobert *et al*. 2004; Przyborski *et al*. 2005) and RIFIN families (Khattab and Klinkert 2006). How the cargo of the MCs is sorted and transported to the surface is unclear. In several mutants, including those lacking REX1 (Dixon *et al*. 2011), SBP1 (Cooke *et al*. 2006; Maier *et al*. 2007), Pf332 (Glenister *et al*. 2009) (although this has been disputed; Hodder *et al*. 2009), MAHRP1 (Spycher *et al*. 2008), PfPTP1 (Rug *et al*. 2014) or several uncharacterized proteins whose genes were deleted in a large-scale study (Maier *et al*. 2008), transport of PfEMP1 to the surface is blocked. Interestingly, in the mutants lacking REX1, SBP1 or Pf332, KAHRP is still transported to the surface, implying that the mutants likely affect a pathway specific for PfEMP1. Several of the uncharacterized proteins that play a part in the transport of PfEMP1 to the surface are conserved in other *Plasmodium* species that do not produce PfEMP1, and therefore must have a role broader than transport of knob-specific cargo (Maier *et al*. 2008). The mechanism of transport of transmembrane proteins from the MCs to the erythrocyte surface remains unclear, although there may be a role for the 25 nm vesicles that have been detected in close proximity to the MCs, as described in more detail below.

In addition, a role for MCs during egress of the parasite has been proposed. Decreasing the expression level of four families of MC resident proteins, PfMC-2TM and three related families, PfEPF1, PfEPF3 and PfEPF4, using a promoter competition approach resulted in an alteration in the release of the merozoites. In contrast to wild-type parasites, mature schizonts in which the expression of these four families was downregulated did not release any additional parasites after an initial release of one to three parasites through the pore that is produced at the time of erythrocyte lysis (Mbengue *et al*. 2013). Hence, the erythrocyte membrane may be modified by these MC resident proteins to facilitate rapid release of the parasites (Glushakova *et al*. 2005; Abkarian *et al*. 2011).

### Essential functions of the MCs

No mutations that result in loss of MCs have been reported and MCs, or organelles very similar to MCs, have been detected in all *Plasmodium* species (Table 1) suggesting that MCs are essential for intraerythrocytic growth, although several mutations lead to altered morphology of MCs, as do certain hemoglobinopathies (Cyrklaff *et al*. 2011). However, of the genes encoding MC proteins that have been targeted only one, *MAHRP2*, has been refractory to deletion (Pachlatko *et al*. 2010). The gene encoding PfD80 is also refractory to deletion (Maier *et al*. 2008), but the initial localization of PfD80 to MCs was indirect (Vincensini *et al*. 2005) and a PfD80-GFP fusion associates with the erythrocyte cytoskeleton, without any indication that it is associated with MCs (Tarr *et al*. 2014). Potentially, the exported proteins encoded by the genes found to be refractory to deletion by Maier *et al*. (2008) may reside in the MC. The localization of MAHRP2 indicates that it may play a part in attachment of the MCs to the cytoskeleton, as described above, which may be essential for the transport of parasite proteins to the erythrocyte surface.

It is not known which parasite proteins transported through MCs are essential for parasite growth *in vitro*. Genes encoding proteins involved in the increased adhesiveness of the infected erythrocyte can be deleted readily, indicating that the increased cellular adhesiveness mediated by PfEMP1 in knobs is not an essential process *in vitro*, although *in vivo* this is likely to be very important for survival in the host. Potentially, the critical factor required for parasite growth *in vitro* that is transported through the MCs functions together with Clag3 to initiate nutrient import. Clag3 does not require the PTEX export system to reach the erythrocyte surface, but is not active on its own, as parasites that lack active HSP101 and hence are unable to export proteins are not sensitive to sorbitol (Beck *et al*. 2014) which requires active PSAC activity (Nguitragool *et al*. 2011). This would fit the observation that Clag3 is present during the ring stage but infected cells do not become sensitive to sorbitol until the trophozoite stage (Saul, Graves and Edser 1990). As mutants that cannot transport PfEMP1 to the surface are viable, the essential factor in the MCs is unlikely to be transported via the same pathway as PfEMP1.

## THE CAVEOLA-VESICLE COMPLEX

In erythrocytes infected with parasites of the *P. vivax*-type (including *P. vivax*, *P. simium* and *P. cynomolgi*) or the *P. ovale*-type (including *P. ovale*, *P. simiovale* and *P. fieldi*), caveoli-like compartments are detected at the plasma membrane of the erythrocyte (Table 1). The first detailed description of these was provided in 1975 by Aikawa and colleagues through EM studies (Aikawa, Miller and Rabbege 1975; Sterling *et al*. 1975). The CVC consists of a cup-shaped invagination of the erythrocyte plasma membrane (a caveola) with a diameter of about 90 nm that is surrounded by small vesicles of about 50 nm. In many cases, the base of the caveola is flattened and marked with an electron dense material, whereas the vesicles are surrounded by small fibrils (Aikawa, Miller and Rabbege 1975). The CVCs of *P. cynomolgi* appear more extensive, with longer extensions than those in *P. vivax* (Aikawa, Miller and Rabbege 1975). Early studies by Tobie and Coatney (1961) using fluorescein-labeled globulins isolated from an individual accidentally infected with *P. cynomolgi bastianellii* showed that parasite-derived antigens were located in a pattern reminiscent of Schüffner dots, and found a similar pattern in *P. vivax*-infected cells. A similar Schüffner-dot-like pattern in *P. vivax*-infected erythrocytes was detected using a monoclonal antibody called A20 in immunofluorescence assays, whereas in EM studies, this antibody labels the CVC (Udagama *et al*. 1988), providing further evidence that the CVCs give rise to the Schüffner dot staining.

The caveola, but not the vesicles, is open to the extracellular surroundings (Aikawa, Miller and Rabbege 1975). EM and immunofluorescence studies showed that the caveola contains parasite antigens (Matsumoto, Aikawa and Barnwell 1988; Barnwell *et al*. 1990), and surface iodination experiments of *P. vivax*-infected erythrocyte showed the presence of a 95 kDa protein on the surface of the infected erythrocyte (Barnwell *et al*. 1990). Determination of the localization of three different antigens, including a 95 kDa protein, using monoclonal antibodies, showed that two of the antigens (95 and a 70 kDa antigen) are present almost exclusively in the CVC, with additional staining in small vesicles in the cytosol. A third protein was detected not only in the CVC, but also in the clefts (Barnwell *et al*. 1990). Localization in two different compartments indicates that transport of proteins to the CVC may be directed through the clefts, although further evidence for the formation of the CVC and its connection to other organelles is lacking. In *P. brasilium*, a 137 kDa antigen present in the CVC was also detected in the micronemes, indicating that CVC proteins are already present during the invasion process and the CVCs are made very soon after invasion of the erythrocyte (Torii *et al*. 1989). Similarly, the 95 kDa antigen is detected in *P. cynomolgi*-infected erythrocytes at very early stages of the infection. This antigen remains the only CVC protein that has been characterized at a molecular level. It was shown to be a member of the PHIST family, a family of exported *Plasmodium* proteins (Matsumoto, Aikawa and Barnwell 1988; Barnwell *et al*. 1990; Sargeant *et al*. 2006; Akinyi *et al*. 2012). Curiously, this protein has orthologs in *P. falciparum* and *P. knowlesi*, two species that do not produce CVCs. It was speculated that a less conserved central region of the protein is responsible for the different localization of the protein in the different species. Attempts to delete the gene encoding this protein in *P. cynomolgi* were unsuccessful, indicating that this protein, and perhaps the CVC, is essential (Akinyi *et al*. 2012).

The function and origin of the CVC remain unclear. It was initially speculated that they were endocytic vesicles derived from host membranes (Aikawa, Miller and Rabbege 1975), although another study suggested that the CVC may be derived from parasite membranes (Sterling *et al*. 1975). The membrane of the caveola binds ferritin, which stains erythrocyte membranes but not parasite membranes, so there is a host component to the CVC (Aikawa, Miller and Rabbege 1975). Erythrocytes infected with *P. vivax* and *P. cynomolgi* are enlarged compared to uninfected erythrocytes, making it tempting to speculate that the CVCs are the conduit through which the additional membrane that allows the erythrocyte to expand is delivered (Sterling *et al*. 1975).

## VESICLES

Two types of vesicles have been detected in the cytosol of infected erythrocytes: 25 nm uncoated vesicles and 80 nm coated vesicles. The 25 nm vesicles are detected almost exclusively near the MCs, whereas the 80 nm vesicles have been detected mostly free in the erythrocyte cytosol.

The 25 nm vesicles are likely to be intimately linked with the MCs and play an important part in the transfer of material from the clefts to the surface of the erythrocyte. EM studies captured the apparent fusion of one of these vesicles with the surface of the erythrocyte plasma membrane (Wickert *et al*. 2003), although no staining with anti-PfEMP1 serum was detected (Hanssen *et al*. 2008b). It was suggested that the latter finding may reflect the small size and hence low number of PfEMP1 molecules, rather that the absence of PfEMP1 in these vesicles.

In contrast to the 25 nm vesicles, the 80 nm vesicles are coated and detected in the erythrocyte cytosol. These vesicles have been detected in the cytosol of infected erythrocytes and become more prominent after treatment with AlF_4_, an activator of G proteins (Crabb *et al*. 1997; Trelka *et al*. 2000; Taraschi *et al*. 2001, 2003; McMillan *et al*. 2013). In those cells, the vesicles, which were 70–100 nm in diameter, were present in chains of coated vesicles. Taraschi *et al*. (2003) detected PfSarp1, PfSec31p, PfEMP1 and PfNSF on these vesicles; the presence of PfEMP1 in these vesicles was subsequently confirmed by McMillan *et al*. (2013). PfSarp1 and PfSec31p had been reported to be present in the erythrocyte cytosol previously (Albano *et al*. 1999; Adisa *et al*. 2002), but more recent reports using GFP fusions revealed that these proteins are present exclusively inside the parasite (Adisa *et al*. 2007). Although the 80 nm vesicles were readily detected in AlF_4_-treated cells, they were rare in untreated cells (Taraschi *et al*. 2003). In contrast, Hanssen *et al*. (2010) detected 80 nm vesicles quite readily when imaging infected erythrocytes using electron tomography and concluded that there are approximately 10 per infected cell. They further noted that these vesicles are absent in the D10 strain, which lacks the genes encoding REX proteins and several other exported proteins. As the MCs in D10 parasites are more stacked, the absence of 80 nm vesicles in this strain may indicate that they are part of the same pathway as the MC formation pathway. Little is known about the cargo of these vesicles. Taraschi and colleagues showed that PfEMP1 is present in the vesicles detected in AlF_4_-treated samples. If these vesicles are related in some way to MC formation, they are likely to contain a plethora of MC proteins, most likely proteins that are destined for the surface of the erythrocyte rather than structural proteins, as these would be expected to be included at the formation of the MC. If this is indeed the case, identification of the cargo could shed light on the modification of the host cell by the parasite through the MC.

## MOBILE COMPARTMENTS AND J DOTS

Whereas the compartments (other than the vesicles) described above are considered to be relatively static, the cytosol of the infected erythrocyte also contains highly mobile compartments. The first visualizations of motile compartments came when infected cells were stained with fluorescent phospholipids (Gormley, Howard and Taraschi 1992). These compartments were initially detected during the early ring stage and were proposed to play a role in the transport of phospholipids, as they were stained with exogenously supplied fluorescent phospholipids. Hibbs and Saul (1994) were the first to characterize motile compartments in detail; using acridine orange, they detected small moving compartments in *P. falciparum*-infected erythrocytes. These were observed mostly during the mid-ring to late-ring stages and appeared to be tethered, despite being fully motile. Fluorescent microscopy did not allow an accurate estimation of their size, but they were estimated to be no larger than 0.1–0.2 μm in diameter. Very similar moving compartments were detected in the cytosol of erythrocytes infected with parasites that produce GFP-labeled PFC0435w, the same GFP-fusion protein that was detected in the neck region of the TVN (van Ooij *et al*. 2008). Similar to the acridine orange-stained vesicles, these motile compartments were detected primarily in the mid-ring stage. Over the course of observation, they did not appear to move in a specific direction and did not accumulate at the periphery of the infected host cell. As this is the same GFP fusion that was detected in the TVN, expressed under the control of the calmodulin promoter, the same caveat about the putative ER-retention signal applies. Endogenous PFC0435w is produced during the trophozoite stage, and therefore it is unlikely to be a normal component of these motile compartments, but could nonetheless serve as a useful marker. In erythrocytes infected with wild-type parasites and stained with Rhodamine B similar motile compartments to those detected in erythrocytes infected with PFC0435w-expressing parasites were detected, indicating that these compartments are not induced by the expression of the transgene (van Ooij *et al*. 2008).

Kulzer *et al*. (2010) have described a population of moving compartments characterized by the presence of the *P. falciparum* proteins PFE0055c and PFA0660w, two type II HSP 40 proteins that had previously been predicted to be exported (Hiller *et al*. 2004; Marti *et al*. 2004; Sargeant *et al*. 2006). As these two proteins contain a J domain, these compartments were named J dots. J dots do not contain the MC markers KAHRP, STEVOR, PfEMP3 and SBP1, indicating that they are distinct compartments, but low levels of PfEMP1 were detected in them. Careful analysis of their velocity revealed that J dots move nearly five times faster than acridine orange-stained compartments, and hence it was concluded that these motile compartments are separate entities. Further supporting this notion is the finding that PFE0055c and PFA0660w are produced throughout the entire intraerythrocytic lifecycle and that the peak fluorescence signal of PFE0055c is seen at the trophozoite stage, different from the mid-ring peak of the acridine orange-positive compartments. Interestingly, both J dots and acridine orange-stained compartments were shown to be expelled from lysing cells, indicating that neither compartment is tightly bound to a cytoskeleton or tethered to the exomembrane system through a membranous connection(Kulzer *et al*. 2010). J dots were not stained with the membrane dye BODIPY ceramide, and thus may not be surrounded by a membrane, although decreasing the cholesterol content of the cell increased the solubility of PFE0055c and PFA0660w. The small size of the J dots may prevent sufficient quantities of dye to be incorporated for visualization. Mobile compartments were also detected by Knuepfer *et al*. (2005) who showed that a PfEMP1 chimera can be seen moving rapidly through the erythrocyte cytosol. Similar fast moving compartments have been detected in *P. chabaudi*; analysis of the localization of a GFP-tagged version of the inhibitor of cysteine proteases revealed a rapid movement of the compartment through the host erythrocyte (Pei *et al*. 2013).

The function of the various motile vesicles is unclear. As the acridine orange-stained compartments are made early and then disappear, they may play a part in the early parts of the rebuilding of the host cell. The presence of PFC0435c in both acridine orange-stained compartments and the TVN may indicate a connection between these two compartments. As acridine orange-stained compartments are present during a time when the MCs are also motile, it is possible that they represent MCs prior to their attachment to the cytoskeleton. In contrast, it has been speculated that J dots transport cholesterol, as the compartments were partly solubilized by the removal of cholesterol (Kulzer *et al*. 2010). The parasite scavenges cholesterol from the host (Maguire and Sherman 1990; Coppens 2013) and sets up a gradient of cholesterol from the erythrocyte plasma membrane to the parasite itself (Tokumasu *et al*. 2014), so without a clear membranous connection between the erythrocyte and the PVM, the pathway for transport of cholesterol could involve proteinaceous carriers in the erythrocyte cytosol. However, this would require host-derived or parasite-derived cholesterol-binding proteins to be part of the J dots, which has not been demonstrated. The presence of PfEMP1 in J dots could alternatively indicate a role in transport of transmembrane proteins. With the discovery or development of additional markers, it should be possible to gain further insight into the function of J dots.

## DISCUSSION

While the exomembrane system is described here as individual compartments (Fig. 1), in all likelihood the various membranous compartments of the *Plasmodium*-infected erythrocyte interact closely and function either together or in some combination of parts. As an example of this, the TVN is contiguous with the PVM, and the two compartment share markers, such as EXP1 and EXP2 (Kara *et al*. 1988; Johnson *et al*. 1994; Lauer *et al*. 1997; Fischer *et al*. 1998). Similarly, the 25 nm vesicles are always detected next to MCs, indicating a possible involvement in the transport of proteins from the clefts to the surface of the erythrocyte. These vesicles are of roughly similar size to the vesicles seen in the CVC, possibly indicating that the CVC may also be involved in transporting proteins and phospholipids to the surface of the erythrocyte. It is striking that a marker of the CVC has orthologs in species in which no CVC has been detected (Akinyi *et al*. 2012).

Some of the reportedly distinct membrane-bound structures described in this review may in fact represent the same compartment. As they have been described using different techniques and different molecular markers, a compartment may appear different in alternate studies. Markers of the MCs are initially present in mobile compartments, which may be the same as, or related to, the acridine orange-stained mobile compartments. These in turn may be identical to the PFC0435w-containing compartments, which, similar to MCs prior to attachment and the acridine orange-stained compartments, are detected in the ring stage, disappear at later stages and are motile. This possibility would be in line with the electron tomographic studies of Hanssen *et al.*(2008b), which provided a detailed look at the infected erythrocyte and did not detect the multitude of different compartments that have been described; a caveat here is that these studies relied on staining with BODIPY ceramide to visualize the internal compartments, and some of the compartments are apparently not visualized by this dye (Kulzer *et al*. 2010). The acridine orange-stained mobile compartments are, however, different from the J dots, as the timing of their presence and their motile velocities are clearly different, but J dots may be the same as the motile compartments that carry PfEMP1, as detected by Knuepfer *et al*. (2005).

**Figure 3.**
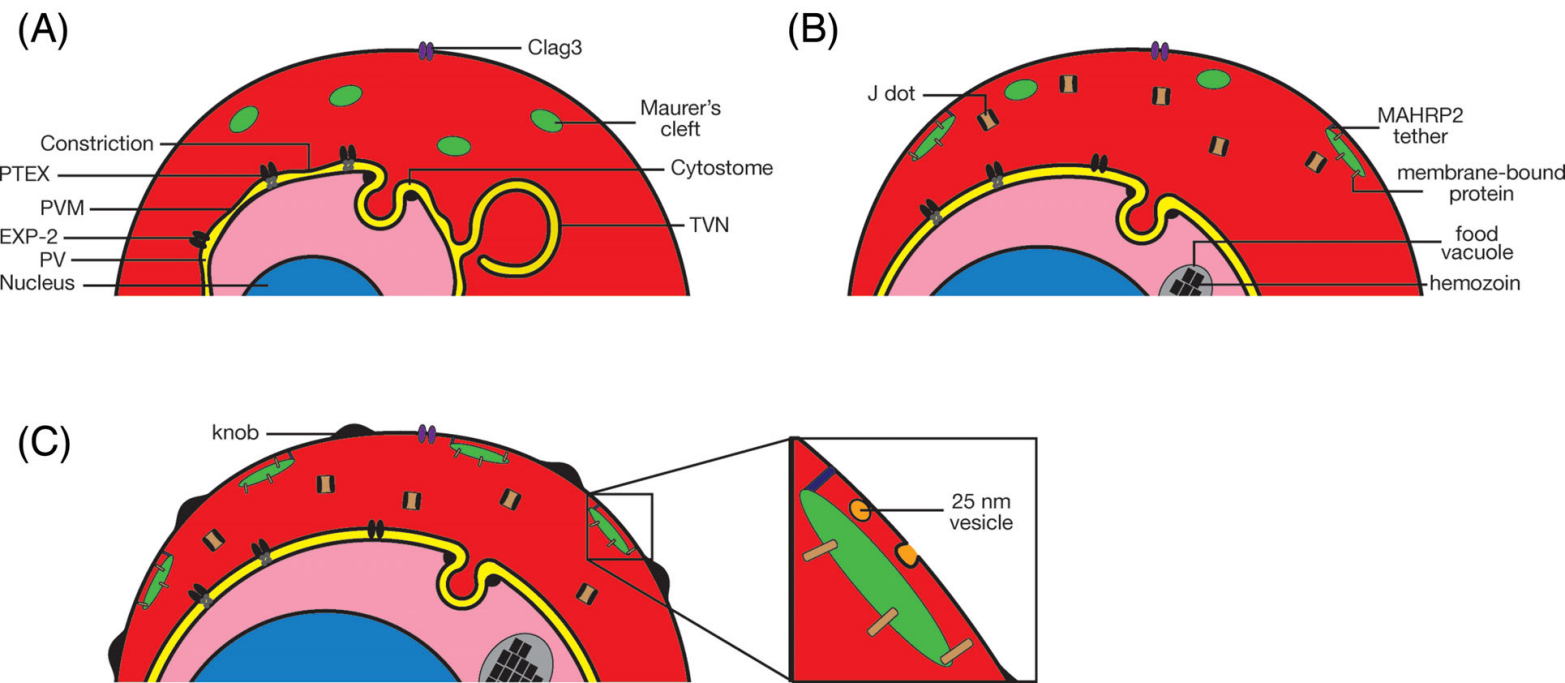
Model of host cell modification through the exomembrane system. (**A**) In newly invaded (young) parasites, the parasite is surrounded by a PVM that contains indentations (forming the ‘necklace of beads’). Protein export and nutrient uptake occurs through EXP2. MCs are still mobile and a prominent TVN is present. Clag3 is exported but nutrient uptake through the PSAC does not yet occur. (**B**) After 20–24 h, hemoglobin uptake through the cytostome initiates and the MCs become tethered to the cytoskeleton or erythrocyte membrane through MAHRP2, allowing transport of membrane proteins from the MC to the surface of the erythrocyte. Transmembrane proteins are transported through the erythrocyte cytosol to the MCs by J dots. Transfer of proteins from the MCs to the erythrocyte surface occurs via 25 nm vesicles. (**C**) As the parasite matures, all the MCs become tethered, knobs are formed and an accessory factor for Clag3 is exported to activate the PSAC.

In a simplified model of the exomembrane system in infected erythrocytes (Fig. 3), the parasite induces formation of the nascent PV during entry of the erythrocyte. This occurs through the invagination of the host membrane, with or without the addition of parasite phospholipids to the host membrane. After the PVM separates from the erythrocyte plasma membrane, further expansion of the PVM is mediated by addition of parasite-derived phospholipids. The mechanism of transfer of these phospholipids to the growing PVM is unclear, but may involve transfer by phospholipid transfer proteins and/or storage of phospholipids in the TVN. In this model, we also propose that EXP2 is released from the parasite soon after invasion and inserts into the PVM to allow for protein export and, based on its ability to complement *T. gondii GRA17* mutants, nutrient uptake, although this has not be experimentally demonstrated in *Plasmodium* spp. Very early after infection, the MCs are formed, resulting in motile compartments that have also been detected as acridine orange-stained compartments and PFC0435w-positive compartments (Fig. 3A). The MCs bind to the cytoskeleton of the host cell during the early trophozoite stage (20–24 h post invasion), possibly through the accumulation of MAHRP2 or synthesis of another parasite protein and the subsequent formation of tethers, and then become immobile (Fig. 3B). The stationary MCs then mediate the transfer of parasite proteins to the host cell, possibly using the 25 nm vesicles to transport cargo proteins to the surface. The timing of the anchoring of the MCs is concomitant with the first appearance of parasite proteins on the surface of the erythrocyte. PfEMP1 synthesis starts at approximately 16 h post-infection (Kyes, Pinches and Newbold 2000), but does not appear on the surface until about 6 h later (Gardner *et al*. 1996). Members of the RIFIN and the STEVOR families, proteins that contain two transmembrane domains, are also transported to the surface of the erythrocyte and are produced at approximately the same time as PfEMP1 (Kyes, Pinches and Newbold 2000). As the MCs are well established at this time, these proteins cannot be inserted into newly forming MCs, but instead require a system for transport through the aqueous environment of the erythrocyte. Movement of J dots, which contain chaperones (Kulzer *et al*. 2012), could be a potential mechanism for this. The heat shock proteins in J dots may play a role in the folding and membrane insertion of transmembrane proteins or the stabilization of these proteins in the absence of a membrane, as no BODIPY-ceramide staining was detected in these compartments. If EXP2 indeed is the portal through which exported proteins with a transmembrane domain cross the PVM, this has important implications for models of the transport of these proteins to the surface of the erythrocyte. The hemolysins to which EXP2 are related are very different transporters than the Sec61 family of transporters that are involved in transport of transmembrane proteins into the ER and the insertion of these proteins into the membrane. In contrast to Sec61, hemolysin E is not known to open to the lipid bilayer to release proteins. Therefore, there is no obvious mechanism by which transmembrane proteins can partition into the lipid bilayer of the PVM during translocation through EXP2 and hence these proteins would be deposited in their entirety into the erythrocyte cytosol. The hydrophobic transmembrane domains would likely require chaperones, either host derived, parasite derived or both, during translocation to the MCs; it is possible that the chaperones in the J dots perform this function (Kulzer *et al*. 2012). There is evidence that PfEMP1, an exported protein with transmembrane spanning regions, is not in a membrane-bound form in the erythrocyte cytosol, giving further credence to the idea that such proteins are transported in a solubilized state (Knuepfer *et al*. 2005; Papakrivos, Newbold and Lingelbach 2005). Investigation of the potential association of additional transmembrane-containing proteins with J dots will allow for further insight into the transport mechanism of this class of proteins. The finding that fast-moving particles are also detected in host cells infected with *P. chabaudi* indicates that these compartments may be involved in protein transport in many species of *Plasmodium* (Pei *et al*. 2013).

One element that is clearly missing from this description of the transport mechanisms in the exomembrane system is a mechanism for fusion and budding of membranes. No known fusion proteins are currently thought to be exported, despite earlier reports (Adisa *et al*. 2001, 2002, 2007; Taraschi *et al*. 2003). It is possible that the host erythrocyte retains sufficient membrane fusion machinery for the parasite to utilize, or that a divergent fusion system is created by the parasite that has escaped detection so far. Alternatively, as suggested in the simplified model (Fig. 3), it is conceivable that after budding of the vesicles that form the MCs soon after entry, no further vesicles are formed (with the exception of the 25 nm vesicles) and proteins, including transmembrane proteins, are transported to the MCs by J dots without the need for vesicle formation.

Many questions remain outstanding regarding the formation of the exomembrane system and the flow of membranes in the infected cell. As membranes and phospholipids are generally more difficult to study than proteins, greater understanding of the membrane system is most likely to come from genetic manipulation of the parasites. With the recent adaptation of the Cas9 system for use in *Plasmodium* parasites (Ghorbal *et al*. 2014; Lee and Fidock 2014; Wagner *et al*. 2014; Zhang *et al*. 2014; de Koning-Ward, Gilson and Crabb 2015) and new systems to regulate gene expression (Pino *et al*. 2012; Collins *et al.*2013a; Prommana *et al*. 2013; Goldfless, Wagner and Niles 2014), the means available to investigate the formation and function of the exomembrane system has greatly improved. Undoubtedly there remain many aspects of the exomembrane system left to be discovered and the resulting insights into the biology of *Plasmodium* infection of its host erythrocyte will be invaluable.
